# A longitudinal analysis of the progression from normal blood pressure to stage 2 hypertension: A 12-year Korean cohort

**DOI:** 10.1186/s12889-020-10115-7

**Published:** 2021-01-06

**Authors:** Eun Sun Yu, Kwan Hong, Byung Chul Chun

**Affiliations:** 1 National Health Insurance Service, Wonju, South Korea; 2 Korea University Graduate School of Public Health, Seoul, South Korea; 3 Department of Preventive Medicine, Korea University College of Medicine, Seoul, 02841 South Korea

**Keywords:** Blood pressure, Incidence, Stage 2 hypertension, Cohort study

## Abstract

**Background:**

The study aimed to estimate the incidence of and period of progression to stage 2 hypertension from normal blood pressure.

**Methods:**

We selected a total of 21,172 normotensive individuals between 2003 and 2004 from the National Health Insurance Service-Health Screening and followed them up until 2015. The criteria for blood pressure were based on the American College of Cardiology/American Heart Association 2017 guideline (normal BP: SBP < 120 and DBP < 80 mmHg, elevated BP: SBP 120–129 and DBP < 80 mmHg, stage 1 hypertension: SBP 130–139 or DBP 80–89 mmHg, stage 2 hypertension: SBP ≥140 or DBP ≥ 90 mmHg). We classified the participants into four courses (Course A: normal BP → elevated BP → stage 1 hypertension→ stage 2 hypertension, Course B: normal BP → elevated BP → stage 2 hypertension, Course C: normal BP → stage 1 hypertension → stage 2 hypertension, Course D: normal BP → stage 2 hypertension) according to their progression from normal blood pressure to stage 2 hypertension.

**Results:**

During the median 12.23 years of follow-up period, 52.8% (*n*= 11,168) and 23.6% (*n*=5004) of the participants had stage 1 and stage 2 hypertension, respectively. In particular, over 60 years old had a 2.8-fold higher incidence of stage 2 hypertension than 40–49 years old. After the follow-up period, 77.5% (*n*=3879) of participants with stage 2 hypertension were found to be course C (*n*= 2378) and D (*n*=1501). After the follow-up period, 77.5% (*n*=3879) of participants with stage 2 hypertension were found to be course C (*n*= 2378) and D (*n*=1501). The mean years of progression from normal blood pressure to stage 2 hypertension were 8.7±2.6 years (course A), 6.1±2.9 years (course B), 7.5±2.8 years (course C) and 3.2±2.0 years, respectively.

**Conclusions:**

This study found that the incidence of hypertension is associated with the progression at each stage. We suggest that the strategies necessary to prevent progression to stage 2 hypertension need to be set differently for each target course.

**Supplementary Information:**

The online version contains supplementary material available at 10.1186/s12889-020-10115-7.

## Background

Hypertension contributes to the global burden of disease [1] and has long been called a silent killer, having no special symptoms during its progression [2]. Due to its asymptomatic characteristic, it is difficult to diagnose and prevent hypertension before its complications occur.

According to the American College of Cardiology/American Heart Association (ACC/AHA) 2017 guideline, the blood pressure target of the criteria for diagnosis of hypertension had changed to a systolic and diastolic blood pressure (BP) of less than 130 mmHg and 80 mmHg, respectively [3]. This aimed to underline the excess risk of BP above this range and to focus public health attention on prevention.

Based on the new guidelines of the ACC/AHA, some studies have been published on the risk factors of hypertension [4], and the prevalence and incidence of hypertension [5]. However, there are no studies that reveal the period of progression from normal blood pressure to stage 2 hypertension (systolic BP ≥140 or diastolic BP ≥ 90 mmHg) and it is unclear whether it progresses gradually from normal BP to stage 2 hypertension passing through all the four stages of BP (normal BP, elevated BP, stage 1 hypertension, stage 2 hypertension) or not.

In addition, BP increases with age in the general population, and the prevalence of secondary hypertension is higher in the older aged groups [6]. However, no large-scale study has been conducted to evaluate the progression from normal BP to stage 2 hypertension in the middle-aged and elderly Korean population.

Therefore, this study aimed to estimate the incidence of and period of progression to stage 2 hypertension from normal blood pressure by age.

## Methods

### Study population

**Fig. 1:**
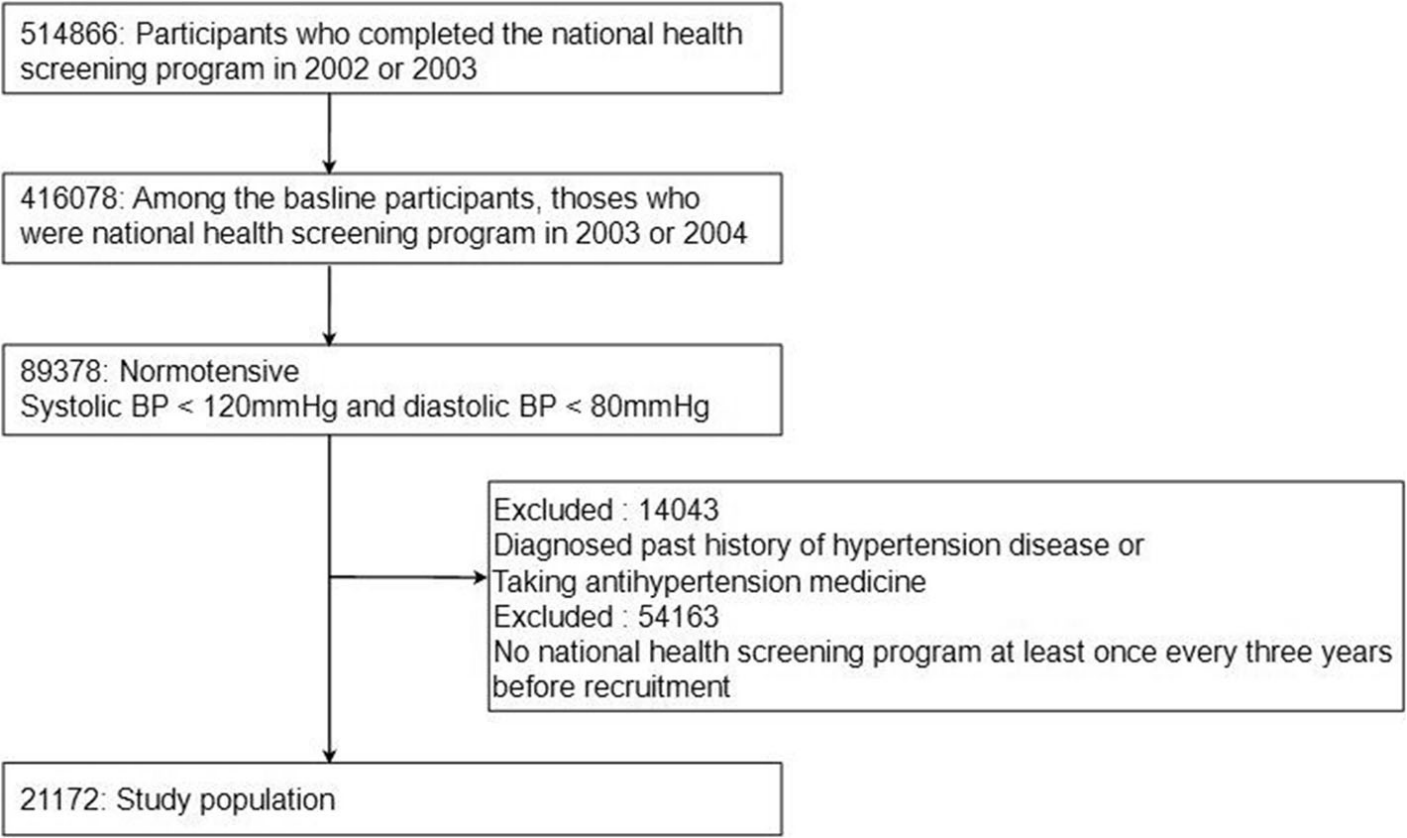
Study flowchart

We used the National Health Insurance Service-Health Screening (NHIS-HealS) in Korea. The NHIS-HEALS consisted of 514,866 participants (aged 40–79 years) randomly selected from 10% of the population from the overall database of the National Health Screening Program between 2002 and 2003 and they were followed-up until December 31, 2015. In the National Health Screening Program, all individuals are invited to participate at least every 2 years in this general, free-of-charge health-screening program. The NHIS-HealS data included information, such as medical diagnoses, drug prescriptions, demographic information, causes of death, and information from health screening test results (biochemical test, health self-questionnaire surveys, family history and physical examinations). Detailed guidance on the cohort has been published previously [7]. From the NHIS-HealS, we selected individuals with normal BP (Systolic BP < 120 mmHg and diastolic BP < 80 mmHg) between 2003 and 2004. Participants were excluded 14,043 if they were receiving an antihypertensive treatment or had a diagnosis of hypertension or had a past history of hypertension between 2002 and 2003. We also excluded 54,163 people who did not participate in the national health-screening program at least once every 3 years. Therefore, 21,172 participants were included in the final analysis. The study flowchart is presented in Fig. 1.

### Classification and measurement of blood pressure

Based on the 2017 ACA/AHA hypertension guidelines [3], this study classified participants into four stages on the basis of the levels of their systolic BP or diastolic BP. The four stages consisted of:

- Normal BP: systolic BP < 120 and diastolic BP < 80 mmHg
- Elevated BP: systolic BP 120–129 and diastolic BP < 80 mmHg
- Stage 1 hypertension: systolic BP 130–139 or diastolic BP 80–89 mmHg
- Stage 2 hypertension: systolic BP ≥140 or diastolic BP ≥ 90 mmHg

Blood pressure was measured using a BP meter after at least 5 min of rest. If the measured value indicated that the systolic BP was < 120 mmHg and the diastolic BP was < 80 mmHg, measured only once. However, If the measured value indicated that the systolic BP was ≥120 mmHg or diastolic BP was ≥80 mmHg, the BP was remeasured after a gap of at least 2 min. The number of re-measurements is three. During the follow-up period, BP measurements were made using the same procedure as at the baseline using the national health screening program. All participants in the study performed health screening program at least once every 3 years for regular blood pressure measurements.

### Definition of the four courses

According to our study, some individuals progressed to stage 2 hypertension with a gradual elevation in blood pressure, while others skipped the elevated BP stage or stage 1 hypertension stage. Therefore, we classified participants into four courses according to their progression from normal BP to stage 2 hypertension until the end of the follow-up in 2015. Participants’ BP level were determined whether to progress to the next stage, depending on the increased BP level in regular health-screening program. However, during the follow-up period, participants diagnosed with stage 2 hypertension in accordance with International Classification of Diseases [ICD-10] (Code I10–I13) were classified as stage 2 hypertension based on that time. As we get older, blood pressure naturally rises, so our research proceeded only in the pre-direction and no one has returned to the previous stage during the follow-up period. In addition, after the final follow-up in 2015, it was classified as normal BP, elevated BP, stage 1 hypertension, and stage 2 hypertension based on maintaining BP levels.

The four courses as follows:

- Course A: normal BP → elevated BP → stage 1 hypertension→ stage 2 hypertension
- Course B: normal BP → elevated BP → stage 2 hypertension
- Course C: normal BP → stage 1 hypertension → stage 2 hypertension
- Course D: normal BP → stage 2 hypertension

### Definition of stage 2 hypertension

In this study, stage 2 hypertension was diagnosed according to the 10th edition of the International Classification of Diseases [ICD-10] (codes I10–I13) or if systolic BP was ≥ 140 mmHg or diastolic BP was ≥ 90 mmHg during a regular health-screening program. The follow-up ended when the participant was diagnosed with stage 2 hypertension, died, or when the study ended (December 31, 2015). During the follow-up, 18 deaths were reported, all of the cases that occurred before death were included in the results. Thus, the mortality during the follow-up period had little affect on the results of this study.

### Statistical analysis

The basic characteristics of the enrolled participants were presented. Continuous variables were expressed as mean and standard deviation (SD) and categorical variables as number and percentage (%). Median follow-up time of study was presented. Mean progression year and SD were presented for each stage of progression as well as for total progression. In addition, we divided the study population into three age groups and evaluated their mean proceeding years representatively. We performed ANCOVA (analysis of covariance) test to determine the differences between the mean proceeding years between the four courses, adjusting for age. Additionally, the time to proceed to each stage was compared between the age groups using the ANOVA (analysis of variance) test. All statistical analyses were performed using SAS Enterprise 7.1 (NHIS remote connection) software and *p*-value < 0.05 were considered statistically significant.

## Results

The general characteristics of the participants according to age groups are presented in Table 1. In this study, participants were divided into three age groups: those in their 40s (age: 40–49 years), 50s (age: 50–59 years), and those aged 60 years and above (age: 60–79 years). In total, 21,172 individuals participated in this study (9690 men and 11,482 women: age 40–79 years). The 40s and 50s groups had a higher proportion of females (55.2 and 55.6%, respectively). The mean ages were 44.6 ±2.3 years (40s group), 51.7±2.0 years (50s group), and 66.1±3.5 years (≥60 years group). Elderly adults (≥60 years of age) had a significantly high frequency of alcohol consumption and had elevated values for systolic BP, fasting blood glucose (FBS), pulse pressure, and aspartate aminotransferase (AST) (*p*< 0.01) than the middle-aged adults (40s and 50s groups). The middle-aged groups had more current smokers and exercised frequently compared to the ≥60 years age group. Family history of hypertension and diabetes was significantly higher in the 40s age group.

**Table 1:** General characteristics of participants by baseline age groups

Variable	Age			
	40-49 years (*n*=17,093)	50-59 years (*n*=25,373)	60–79 years (*n*=1542)	P for Trend^a^
Age (years)	44.6±2.3	51.7±2.0	66.1±3.5	< 0.01
Sex				
Male	7111 (45.8)	1776 (44.4)	803 (48.9)	0.29
Female	8416 (55.2)	2227 (55.6)	839 (51.1)	
Systolic BP (mm Hg)	106.3±7.2	106.6±7.1	107.1±7.2	< 0.01
Diastolic BP (mm Hg)	66.6±5.9	67.2±5.9	66.6±6.1	< 0.01
Body mass index (kg/m^2^)	22.8±2.5	23.1±2.5	22.7±2.8	< 0.01
Fasting glucose (mg/dl)	90.4±24.0	91.5±18.8	93.7±25.2	< 0.01
Pulse pressure (mm Hg)	39.7±6.3	39.7±6.3	40.5±6.8	< 0.01
Total cholesterol (mg/dl)	188.7±33.7	198.0±35.6	197.3±36.0	< 0.01
Aspartate aminotransferase (U/L)	23.0±15.8	24.4±12.7	26.2±17.7	< 0.01
Alanine aminotransferase (U/L)	21.6±19.2	22.9±22.0	21.4±11.7	< 0.01
Gamma GTP (U/L)	25.1±27.6	26.6±26.8	24.7±31.7	< 0.01
Hemoglobin (g/dl)	13.6±1.6	13.6±1.4	13.5±1.4	< 0.01
Smoking status				
No	11,880 (79.1)	3121 (81.1)	1312 (82.8)	< 0.01
Current	3138 (20.9)	725 (18.9)	272 (17.2)	
Drinking frequency				
1–2/week	14,423 (94.2)	3657 (93.2)	1431 (89.4)	< 0.01
3–5/week	882 (5.8)	268 (6.8)	169 (10.6)	
Exercise frequency				0.19
not at all	12,160 (79.9)	3103 (79.3)	1315 (82.2)	
3–5/ week	3059 (20.1)	808 (20.7)	285 (17.8)	
History of diabetes				< 0.01
No	15,358 (98.9)	3921 (98.0)	1578 (96.1)	
Yes	169 (1.1)	82 (2.1)	64 (3.9)	
Family history of hypertension				< 0.01
No	13,106 (93.0)	3406 (93.7)	1406 (96.6)	
Yes	979 (7.0)	231 (6.3)	50 (3.4)	
Family history of diabetes				< 0.01
No	12,945 (91.8)	3368 (92.6)	1405 (96.5)	
Yes	1158 (8.2)	268 (7.4)	51 (3.5)	

Values are expressed as mean ± SD (standard deviation) or number (percentage)

^a^P for trend: Cochran-Armitage test for categorical variables and ANOVAs for continuous variables

**Fig. 2:**
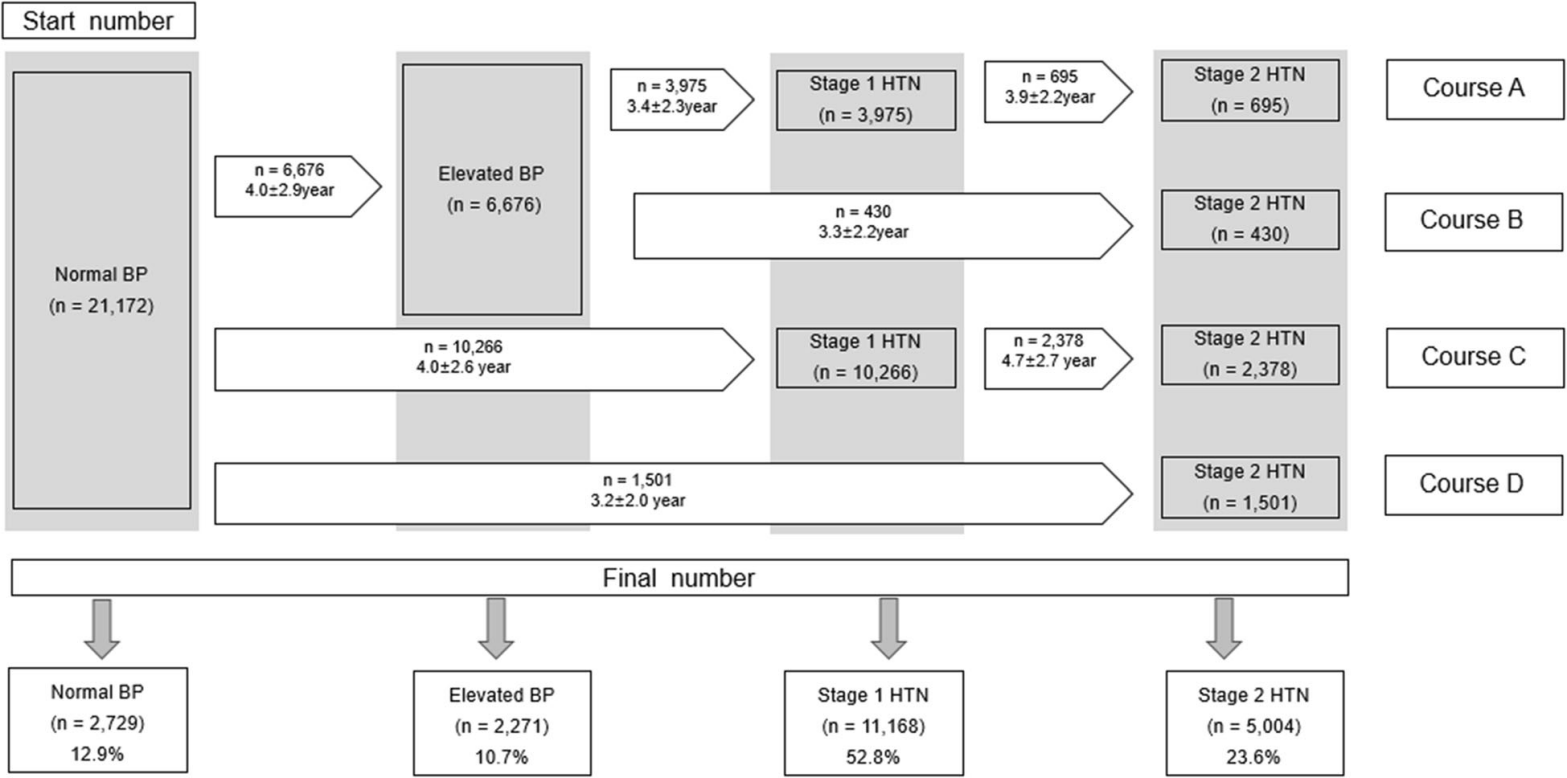
The progression from normal BP to stage 2 hypertension in the total study population

Figure 2 shows the four courses of progression from normal BP to stage 2 hypertension of all participants. During the follow-up period, the combined incidence of stage 1 and stage 2 hypertension was 76.4% (*n*= 16,172). Among them, 23.6% (*n*= 5004) of the participants had stage 2 hypertension and 52.8% (*n*= 11,168) had stage1 hypertension. Only 12.9% (*n*= 2729) of the participants maintained normal BP.

After the follow-up period, 77.5% (*n*= 3879) of participants with stage 2 hypertension were found to be in courses C (*n*= 2378) and D (*n*= 1501). They had a 3.4-fold higher incidence of stage 2 hypertension than courses A (*n*= 695) and B (*n*= 430). The difference between courses C and D and courses A and B is that course C and D skipped the elevated BP stage and progressed directly to stage 2 hypertension. Additionally, more participants directly progressed from normal BP to stage 1 hypertension (*n*= 10,266) than the number of participants who progressed from normal BP to elevated BP (*n*= 6676).

The study showed similar results when participants were divided into different age groups (age: 40–49/50–59/60–79), sex (male/female) and body mass index (BMI,< 25/≥25 kg/m^2^). Progression by age groups, sex and BMI can be found in the supplementary Figures 1, 2, 3, 4 and 5.

Table 2 shows the adjusted mean years and median years from normal BP to stage 2 hypertension for participants by courses. The adjusted mean years and median years of progression from normal BP to stage 2 hypertension were 8.6±2.6 years and 8.97 years (course A), 6.1±2.9 years and 5.65 years (course B), 7.5±2.8 years and 7.61 years (course C) and 3.2±2.0 years and 2.34 years (course D). Participants in course D had the shortest duration of progression to stage 2 hypertension, while participants in course A had a longer duration of progression to stage 2 hypertension. Those who skipped any middle stage of progression from normal BP to stage 2 hypertension were rapidly progressed to stage 2 hypertension.

**Table 2:** Adjusted mean years and median years from normal BP to stage 2 hypertension for participants by courses

Results	Mean progression years from normal BP to stage 2 HTN (mean±SD)	Median progression years from normal BP to stage 2 HTN	*P*-value
Course A (*n*=695)	8.6±2.6	8.97	< 0.01
Course B (*n*=430)	6.1±2.9	5.65	
Course C (*n*=2378)	7.5±2.8	7.61	
Course D (n=1501)	3.2±2.0	2.34	

The *p*-value for ANCOVA (analysis of covariance) test adjusting for age is represented

*BP* blood pressure, *HTN* hypertension

Course A: normal BP → elevated BP → stage1 HTN → stage2 HTN

Course B: normal BP → elevated BP → stage2 HTN

Course C: normal BP → stage1 HTN → stage2 HTN

Course D: normal BP → stage2 HTN

Table 3 shows the participants BP category after 12 years follow-up by baseline age groups. There were differences in the proportion of blood pressure levels by age groups in all final stages (p for trend < 0.01). During the 12-year follow-up period, progression to stage 2 hypertension was observed for 5004 participants (23.6%). The high incidence of stage 2 hypertension was associated with older age, male sex and higher BMI.

**Table 3:** Participants BP category after 12 years follow-up by baseline age groups

Results	Remained as normal BP	Remained as elevated BP	Remained as stage 1 HTN	Resulted as stage 2 HTN
40–49 years (*n*=15,527)	2204 (14.2)	1726 (11.1)	8566 (55.2)	3031 (19.5)
50–59 years (*n*=4003)	452 (11.3)	457 (11.4)	2024 (50.6)	1070 (26.7)
60–79 years (*n*=1642)	73 (4.5)	88 (5.4)	578 (35.2)	903 (55.0)
Total (*n*=21,172)	2729 (12.9)	2271 (10.7)	11,168 (52.8)	5004 (23.6)

Values are expressed as number and percentage (%)

There were differences in the proportion of blood pressure levels by age groups in all final stages (p for trend < 0.01)

*BP* blood pressure, *HTN* hypertension

Table 3 shows the results by age group. After the final follow-up, 14, 11 and 4% of the participants maintained a normal BP, in the 40s, 50s and ≥60 years age groups, respectively. The number of participants in the 40s and 50s groups who maintained with elevated BP was similar (40s: 11.1%, 50s: 11.4%), while that in the ≥60 years age group was much lower at 5.4%. Among the 40s and 50s age groups, highest number of participants progressed to stage 1 hypertension (> 50%) while 55.0% of the ≥60 years age group progressed to stage 2 hypertension. In particular, the incidence of stage 2 hypertension more than doubled among participants above 60 years of age than 40s and 50s.

Supplementary Tables 1 and 2 show the results by sex and BMI. After the final follow-up, The number of female participants who maintained with normal BP or elevated BP were 29.9%, while that in male was more lower at 16.2%. However, the incidence of stage 2 hypertension higher male (26.5%) than female (21.2%). 14.4% of those with normal BMI (< 25 kg/m^2^) maintained normal BP, while only 6.5% of those with high BMI (≥25 kg/m^2^). Participants with high BMI had a high incidence of stage 2 hypertension of 34.0%, while those with normal BMI had a low incidence of stage 2 hypertension of 21.2%. The results of sex and BMI can be found in the supplemental material.

Table 4 shows the comparison of the period of progression by age groups from normal to elevated BP, stage 1 hypertension, or stage 2 hypertension. The duration of the progression from normal BP to elevated BP or stage 2 hypertension had a significant difference between the groups (≥60 years vs 40s–50s) (*p*< 0.01). The period of progression from normal BP to elevated BP or stage 2 hypertension was similar among the 40s and 50s groups. However, the ≥60 years age group (2.8±1.6 year) had a much shorter period of progression to stage 2 hypertension than the 40s–50s groups (3.6±2.3 year and 3.5±2.3 year).

**Table 4:** Comparison of the period of progression by age groups: from normal to elevated BP, stage 1 HTN, or stage 2 HTN

Course	Age (mean±SD)			*P*-value
	40–49 years (*n*= 13,323)	50–59 years (*n*= 3551)	60–79 years (*n*= 1580)	
Normal BP→ Elevated BP	4.0±3.0 5031 (37.8)	4.1±3.0 1237 (34.9)	3.6±2.3 408 (25.8)	0.01^a^
Normal BP→ Stage 1 HTN	4.1±2.7 7442 (55.9)	3.9±2.6 1980 (55.8)	3.5±2.3 855 (54.1)	< 0.01^b^
Normal BP→ Stage 2 HTN	3.6±2.3 850 (6.4)	3.5±2.3 334 (9.4)	2.8±1.6 317 (20.1)	< 0.01^a^

Values are expressed as number and percentage (%)

*P*-values are from ANOVA test with Duncan method of mean proceed time between age groups

*BP* blood pressure, *HTN* hypertension

^a^Two age groups (40–49 years and 50–59 years) were different with 60–79 years

^b^All three groups were statistically different

The duration of the progression from normal BP to stage 1 hypertension was different in the three groups. As a result, the mean duration of progress of all stages to stage 2 hypertension was shortest in the ≥60 years age group.

## Discussion

We examined the incidence of and the progression to stage 2 hypertension in normotensive individuals in a longitudinal cohort study with over 12 years of follow-up. The risk of hypertension is well known in past studies [8, 9], but the progression and risk of hypertension at each stage of BP assessment has not been well quantified.

To the best of our knowledge, there is currently no long-term study that details the progression from normal BP to stage 2 hypertension by age. This study presents the natural history of stage 2 hypertension progression. Previous studies about the natural history of hypertension have connected childhood BP levels with cardiovascular events in later adulthood and prehypertension with cardiovascular risk factors [10, 11].

Among the 21,172 participants with normotension, we identified 5004 (23.6%) participants with progression to stage 2 hypertension during the 12-year follow-up period. During the follow-up period, the proportion of stage 2 hypertension among participants in course C and D (76.4%) was much higher compared to that in courses A and B (23.6%). Participants who skipped the elevated BP stage, such as those in courses C and D, had a much higher risk of developing stage 2 hypertension. Additionally, more participants directly progressed from normal BP to stage 1 hypertension (*n*=10,266) than participants who progressed from normal BP to elevated BP (*n*=6676). This means that many participants progressed to stage 1 hypertension, skipping the elevated BP stage.

Age is a major predisposing factor for most common degenerative diseases and increasing age is a well-known and important risk factor for hypertension [12–14]. The Asia cohort study revealed that the incidence of hypertension in older age (> 55 years) was higher than in younger age (20–54 years) [15]. This study showed that after the follow-up period, the incidences of stage 2 hypertension were 19.5% (40s group), 26.7% (50s group), and 55.0% (≥60 years age group). The ≥60 years age group had a 2.8-fold higher incidence of stage 2 hypertension than the 40s group. The Framingham study [16] showed that the incidence of cardiovascular disease increased gradually as systolic BP increased with age. This means that not only middle-aged but also elder adults should consider hypertension management. This study has considerable significance because we presented the mean years of progression for all the courses, using adjusted age ANCOVA analysis.

The incidence of hypertension in individuals without hypertension is likely to vary depending on the initial BP value, the variation of BP measurements, the tracking period, and the presence of factors predisposing to hypertension [17]. The results of the present study revealed that the incidence of stage 2 hypertension was low when progressing through all the stages of BP, but high when any middle stage was skipped. The adjusted mean years of progression from normal BP to stage 2 hypertension were 8.6±2.6 years (course A), 6.1±2.9 years (course B), 7.5±2.8 years (course C) and 3.2±2.0 years (course D). In addition, elderly adults had shorter progress periods to stage 2 hypertension than middle-aged adults. Individuals with high BP (systolic BP 120–139 mmHg or diastolic BP 80–89 mmHg), frequently progressed to hypertension over a period of 4 years, especially older adults [18]. Therefore, to detect the onset of hypertension, we recommend frequent BP screenings for individuals ≥60 years of age with normal or elevated BP.

### Strengths and limitations

There are several limitations to this study. The National Health Screening Program was performed in hospitals using standard measurement criteria, and there might have been differences in the measurement times and BP meters. However, the Framework Act on the National Health Examination requires health examination hospitals to complete quality assessments for BP measuring instruments every 3 years. Secondly, the four courses we have presented are not universal because they are based on the BP category presented in this study. In addition, the meaning of the “skipped stages” used in this study means that the stage of blood pressure progression was divided into four stages (normal BP, elevated BP, stage 1 hypertension, stage 2 hypertension) and did not go through the next stage during blood pressure progression. It is unclear whether the participants of the study actually skip the next stage or whether it is a process of slow progression. Therefore, further study will be needed on whether the participants actually skipped next stage or the slow progress rate process. However, the result can be of sufficient value since this was the first attempt to verify the natural four natural courses from normal blood pressure to stage 2 hypertension.

## Conclusions

This study emphasized the increased risk of hypertension with age and found that the incidence of hypertension is related not only to age but also to the progression of each BP stage. In addition, in order to prevent progress into stage 2 hypertension, different systematic prevention strategies are needed according to the four courses, not uniform hypertension prevention education. In particular, senior citizens or those who have progressed directly from normal BP to stage 1 hypertension need to be warning and preventive education for stage2 hypertension, and BP screening should be conducted frequently. Furthermore, our results underscore the need for further studies of the determinants to predict who will go for hypertension in which course.

## Supplementary Information

**Additional file 1: Supplementary Figure 1.** The progression from normal BP to stage 2 hypertension (Ages: 40-49/50-59/60-79). **Supplementary Figure 2.** The progression from normal BP to stage 2 hypertension (Sex: male). **Supplementary Figure 3.** The progression from normal BP to stage 2 hypertension (Sex: female). **Supplementary Figure 4.** The progression from normal BP to stage 2 hypertension (BMI<25 kg/m^2^). **Supplementary Figure 5.** The progression from normal BP to stage 2 hypertension (BMI≥25 kg/m^2^)

**Additional file 2: Supplemental table 1.** Participants BP category after 12 years follow-up by sex. **Supplemental table 2.** Participants BP category after 12 years follow-up by body mass index

## Data Availability

The data were obtained from the National Health Insurance Sharing Service by National health insurance company in Korea. Due to legal restrictions, the database cannot be made publicly available. However, data are available from the authors upon reasonable request and with permission of the National Health Insurance Sharing Service.

## Abbreviations

NHIS-HealS: National Health Insurance Service-Health Screening
ACC/AHA: American College of Cardiology/American Heart Association;
BP: Blood pressure
SBP: Systolic blood pressure
DBP: Diastolic blood pressure
ICD-10: International Classification of Diseases
ANCOVA: Analysis of covariance
SD: Standard deviation
HTN: Hypertension

## References

[1] Kearney PM, Whelton M, Reynolds K, Muntner P, Whelton PK, He J (2005). Global burden of hypertension: analysis of worldwide data. Lancet.

[2] Moore J (2005). Hypertension: catching the silent killer. Nurse Pract.

[3] Carey RM, Whelton PK (2018). Prevention, detection, evaluation, and management of high blood pressure in adults: synopsis of the 2017 American College of Cardiology/American Heart Association Hypertension Guideline. Ann Intern Med.

[4] Huang Y, Dai M, Deng Z, Huang X, Li H, Bai Y, et al. Clustering of risk factors and the risk of new-onset hypertension defined by the 2017 ACC/AHA Hypertension Guideline. J Hum Hypertens. 2019:1–6. 10.1038/s41371-019-0232-9. 10.1038/s41371-019-0232-9 31431682

[5] Kim T-J, Lee J-W, Kang H-T, Cho M-C, Lim H-J, Kim J-Y (2018). Trends in Blood Pressure and Prevalence of Hypertension in Korean Adults Based on the 1998–2014 KNHANES. Yonsei Med J.

[6] Anderson GH (1999). Effect of age on hypertension: analysis of over 4,800 referred hypertensive patients. Saudi J Kidney Dis Transpl.

[7] Kim Y, Han B-G, Group K (2017). Cohort profile: the Korean genome and epidemiology study (KoGES) consortium. Int J Epidemiol.

[8] Kishore J, Gupta N, Kohli C, Kumar N. Prevalence of hypertension and determination of its risk factors in rural Delhi. Int J Hypertens. 2016;6. 10.1155/2016/7962595. 10.1155/2016/7962595 PMC4834167 27127646

[9] Bosu WK, Aheto JM, Zucchelli E, Reilly S (2017). Prevalence, awareness, and associated risk factors of hypertension in older adults in Africa: a systematic review and meta-analysis protocol. Syst Rev.

[10] Falkner B (2010). Hypertension in children and adolescents: epidemiology and natural history. Pediatr Nephrol.

[11] Pannarale G, Moroni C, Acconcia M, Pannitteri G, Truscelli G, Valente L (2017). The natural history of prehypertension. A 20-year follow-up. Eur Rev Med Pharmacol Sci.

[12] Bavishi C, Goel S, Messerli FH (2016). Isolated systolic hypertension: an update after SPRINT. Am J Med.

[13] Poorolajal J, Farbakhsh F, Mahjub H, Bidarafsh A, Babaee E (2016). How much excess body weight, blood sugar, or age can double the risk of hypertension?. Pub Health.

[14] Hong K, Yu ES, Chun BC (2020). Risk factors of the progression to hypertension and characteristics of natural history during progression: A national cohort study. Plos one.

[15] Prabhakaran D, Jeemon P, Ghosh S, Shivashankar R, Ajay VS, Kondal D (2017). Prevalence and incidence of hypertension: results from a representative cohort of over 16,000 adults in three cities of South Asia. Indian Heart J.

[16] Vokonas P, Kannel W, Cupples L (1988). Epidemiology and risk of hypertension in the elderly: the Framingham study. J Hypertens Suppl.

[17] Vasan RS, Larson MG, Leip EP, Kannel WB, Levy D (2001). Assessment of frequency of progression to hypertension in non-hypertensive participants in the Framingham Heart Study: a cohort study. Lancet.

[18] Chobanian AV, Bakris GL, Black HR, Cushman WC, Green LA, Izzo JL (2003). Seventh report of the joint national committee on prevention, detection, evaluation, and treatment of high blood pressure. Hypertension.

