# Supplementary material for: A longitudinal analysis of the progression from normal blood pressure to stage 2 hypertension: A 12-year Korean cohort
Source: BMC Public Health. 2021 Jan 6;21:61. doi: 10.1186/s12889-020-10115-7 (PMC7788775; doi:10.1186/s12889-020-10115-7)
Supplement: Supplementary file 1 — Additional file 1: Supplementary Figure 1. The progression from normal BP to stage 2 hypertension (Ages: 40-49/50-59/60-79). Supplementary Figure 2. The progression from normal BP to stage 2 hypertension (Sex: male). Supplementary Figure 3. The progression from normal BP to stage 2 hypertension (Sex: female). Supplementary Figure 4. The progression from normal BP to stage 2 hypertension (BMI<25 kg/m2). Supplementary Figure 5. The progression from normal BP to stage 2 hypertension (BMI≥25 kg/m2) [file 12889_2020_10115_MOESM1_ESM.pdf]

SUPPLEMENTAL MATERIAL

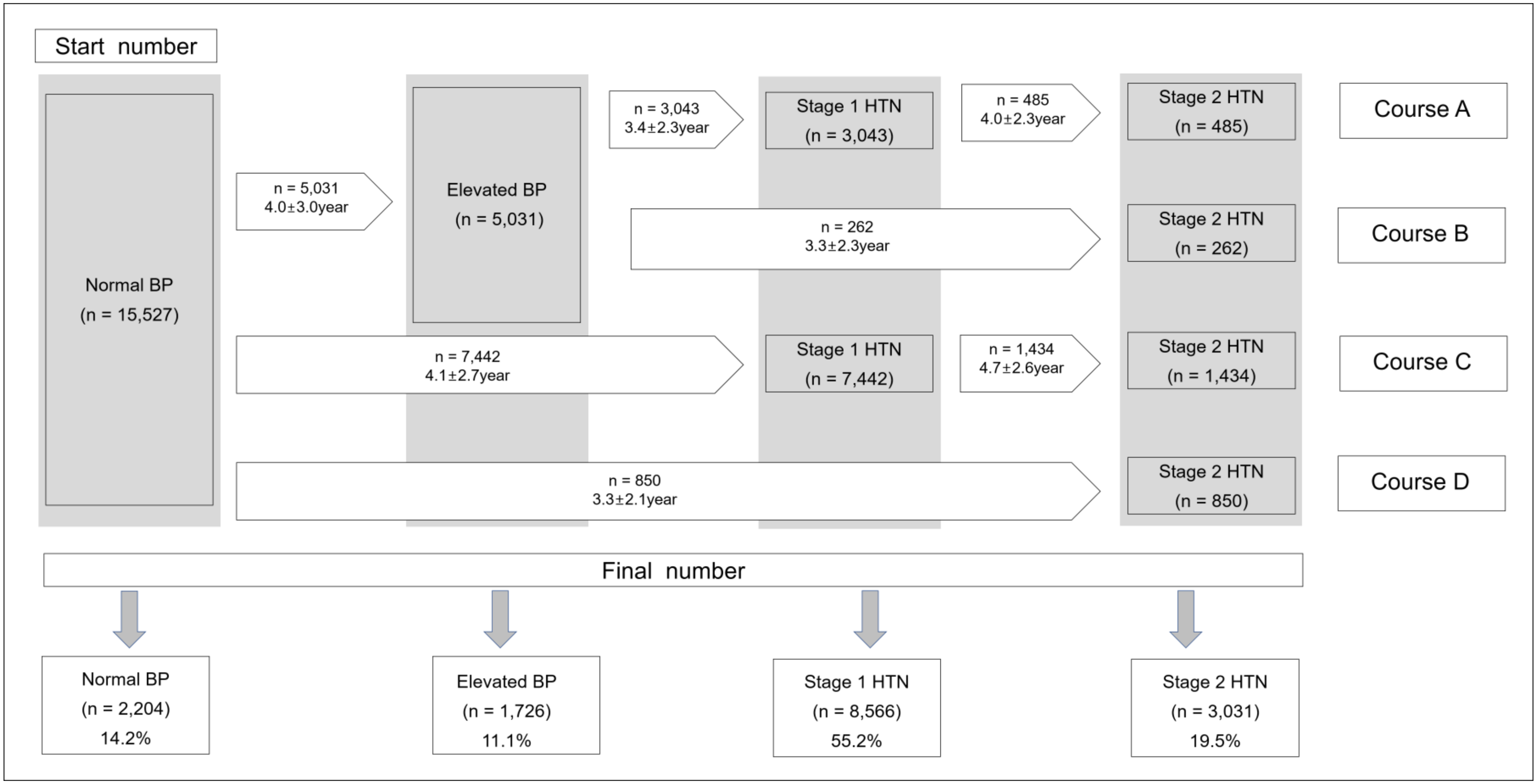

Values are expressed as n, mean ±SD (standard deviation) year. BP = blood pressure, HTN = hypertension (Ages: 40-49)

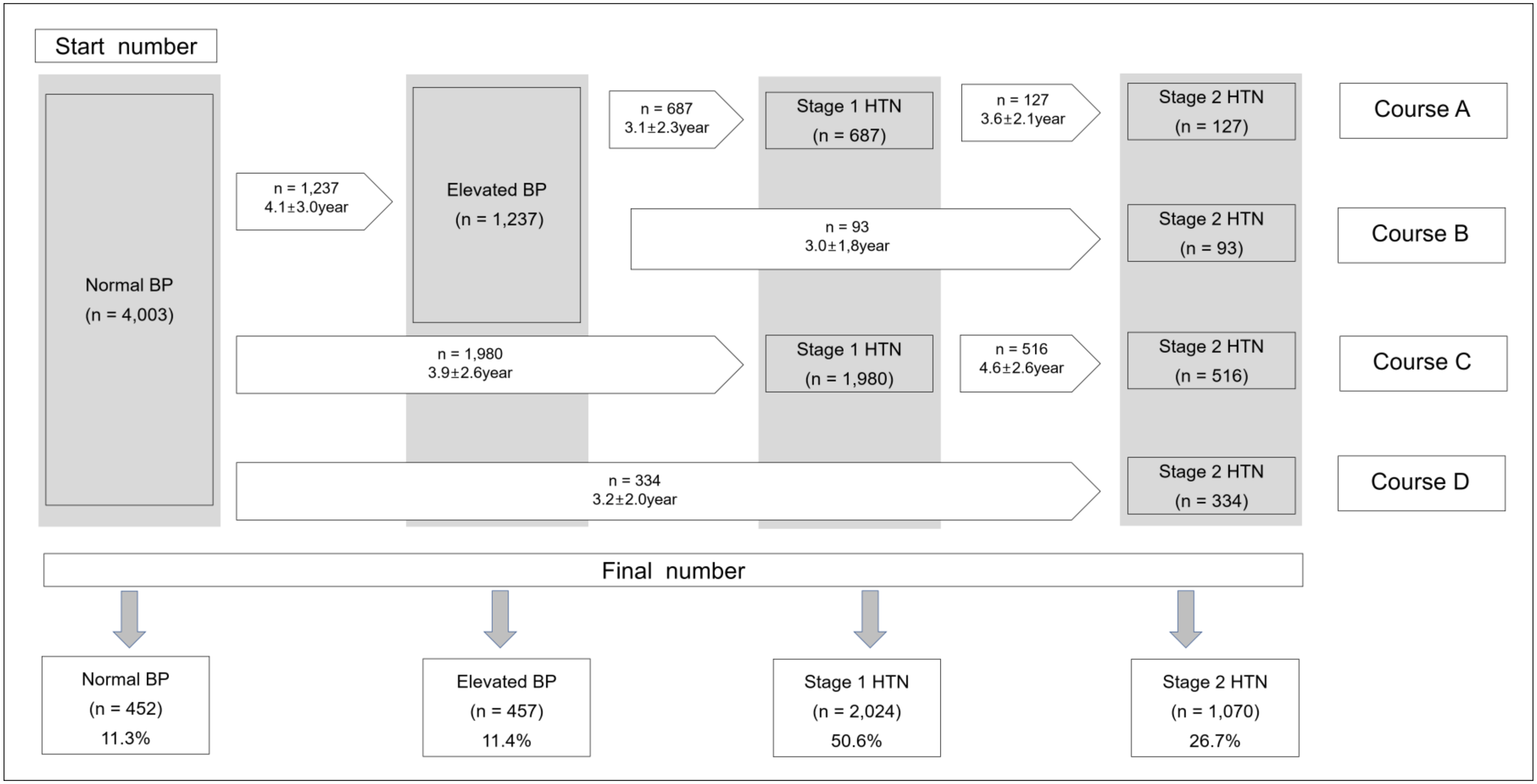

Values are expressed as n, mean ±SD (standard deviation) year. BP = blood pressure, HTN = hypertension (Ages: 50-59)

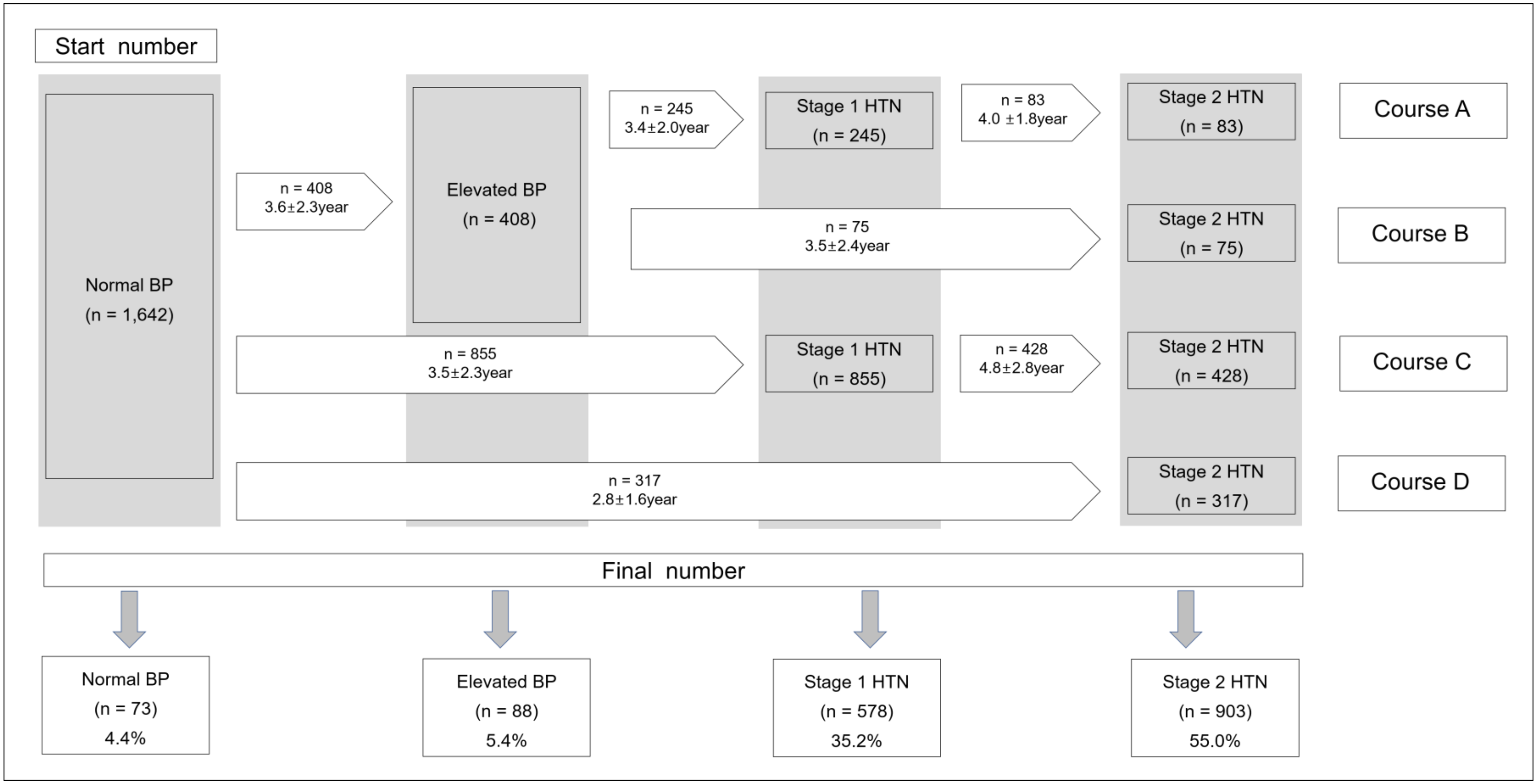

Values are expressed as n, mean ±SD (standard deviation) year. BP = blood pressure, HTN = hypertension (Ages: 60-79)

Supplemental figure 1. The progression from normal BP to stage2 hypertension (Ages: 40-49/50-59/60-79)

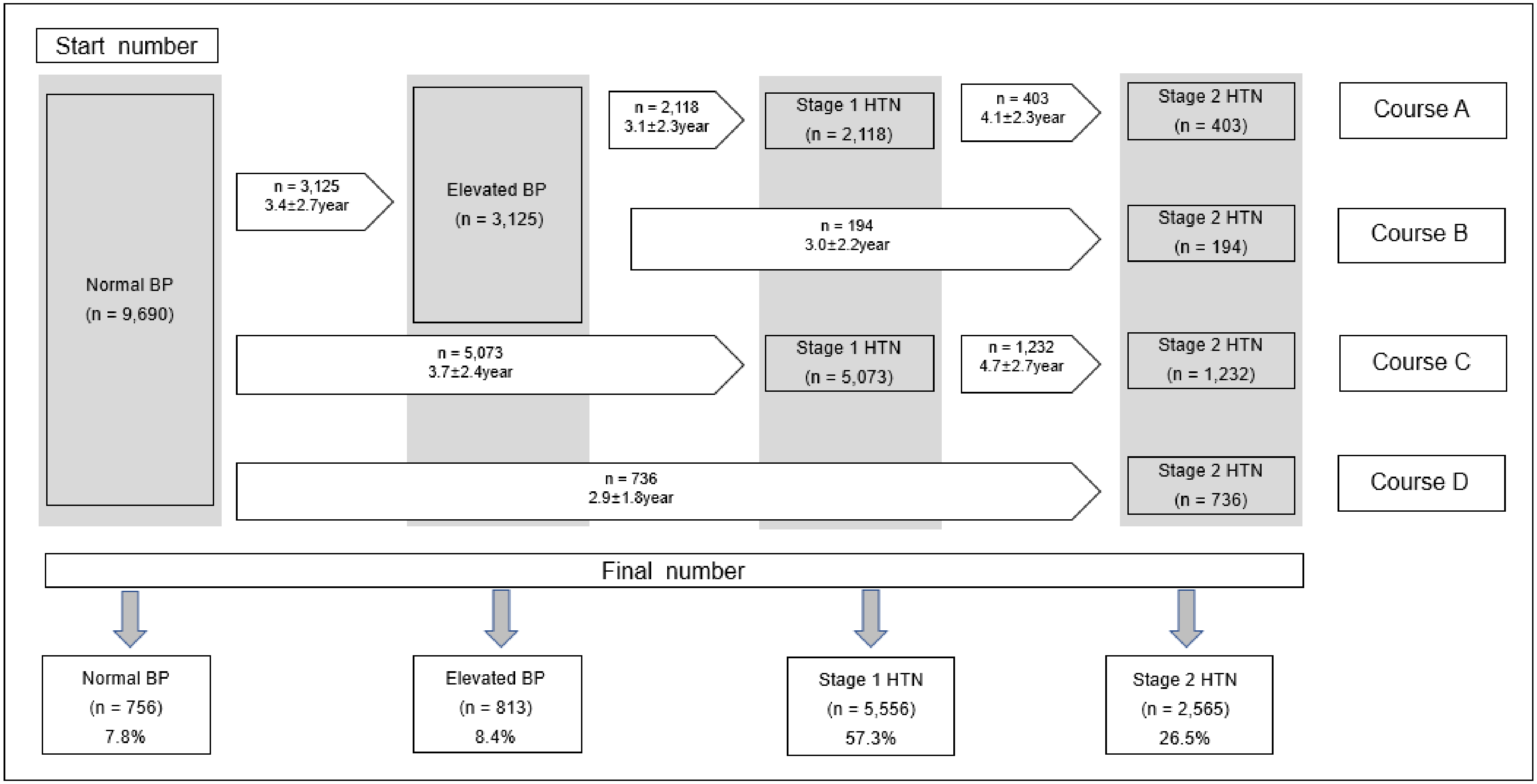

Values are expressed as n, mean ±SD (standard deviation) year. BP = blood pressure, HTN = hypertension

Supplemental figure 2. The progression from normal BP to stage2 hypertension (Sex: male)

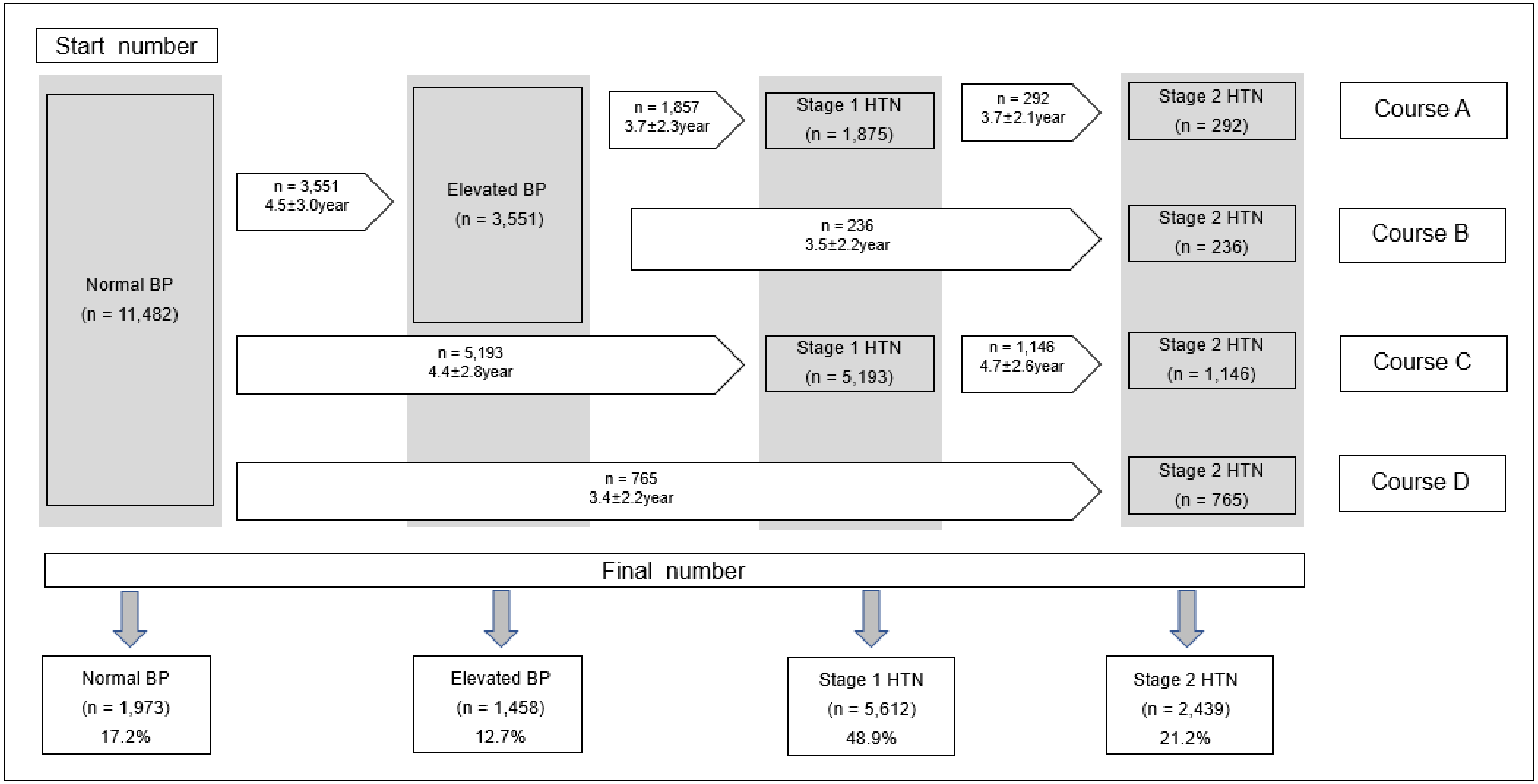

Values are expressed as n, mean ±SD (standard deviation) year. BP = blood pressure, HTN = hypertension

Supplemental figure 3. The progression from normal BP to stage2 hypertension (Sex: female)

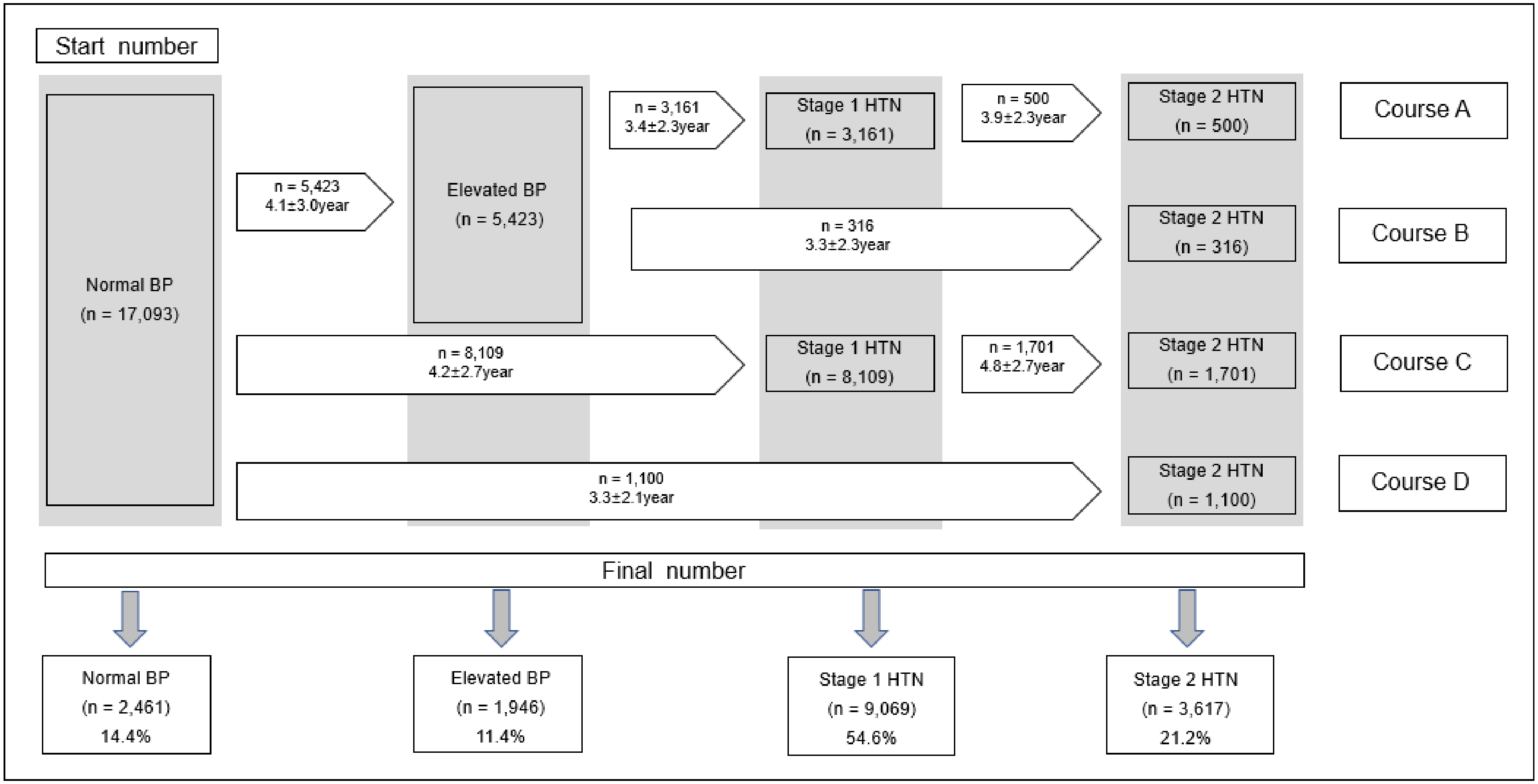

Values are expressed as n, mean ±SD (standard deviation) year. BP = blood pressure, HTN = hypertension

Supplemental figure 4. The progression from normal BP to stage2 hypertension (BMI<25 kg/m^2)

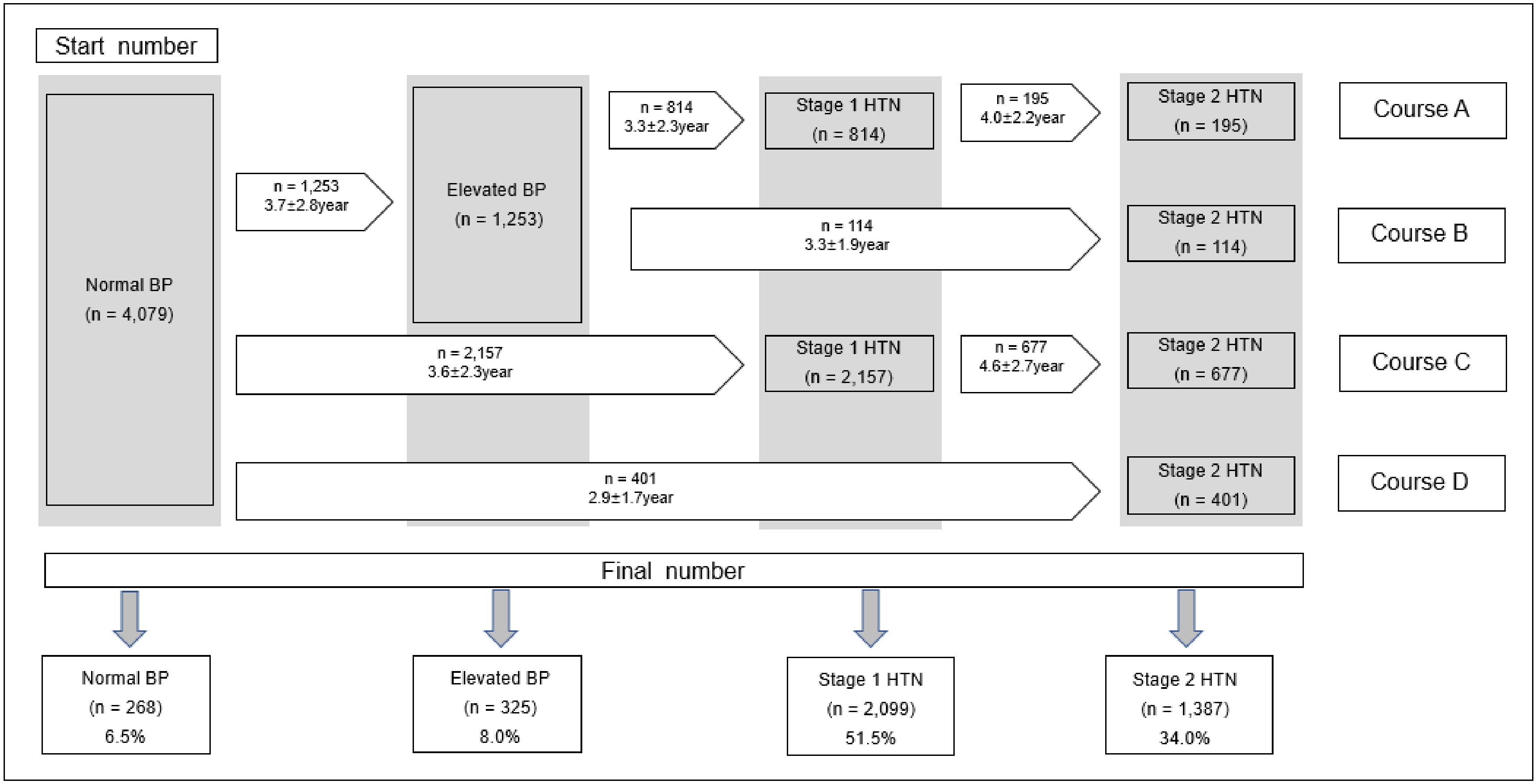

Values are expressed as n, mean ±SD (standard deviation) year. BP = blood pressure, HTN = hypertension

Supplemental figure 5. The progression from normal BP to stage2 hypertension (BMI≥25 kg/m^2)
