# Supplementary material for: A longitudinal analysis of the progression from normal blood pressure to stage 2 hypertension: A 12-year Korean cohort
Source: BMC Public Health. 2021 Jan 6;21:61. doi: 10.1186/s12889-020-10115-7 (PMC7788775; doi:10.1186/s12889-020-10115-7)
Supplement: Supplementary file 2 — Additional file 2: Supplemental table 1. Participants BP category after 12 years follow-up by sex. Supplemental table 2. Participants BP category after 12 years follow-up by body mass index [file 12889_2020_10115_MOESM2_ESM.docx]

**SUPPLEMENTAL MATERIAL**

**Supplemental table 1. Participants BP category after 12 years follow-up by sex**

| Results | Remained as  normal BP | Remained as  elevated BP | Remained as  stage 1 HTN | Remained as  stage 2 HTN |
| --- | --- | --- | --- | --- |
| Male  (n=9690) | 756 (7.8) | 813 (8.4) | 5556 (57.3) | 2565 (26.5) |
| Female  (n=11482) | 1973 (17.2) | 1458 (12.7) | 5612 (48.9) | 2439 (21.2) |
| Total  (n=21172) | 2729 (12.9) | 2271 (10.7) | 11168 (52.8) | 5004 (23.6) |

Values are expressed as number and percentage (%).

There were differences in the proportion of blood pressure levels by sex in all final stages (p-value<0.01).

BP = blood pressure, HTN = hypertension

**Supplemental table 2. Participants BP category after 12 years follow-up by body mass index**

| Results | Remained as  normal BP | Remained as  elevated BP | Remained as  stage 1 HTN | Remained as  stage 2 HTN |
| --- | --- | --- | --- | --- |
| <25kg/m^2^  (n=17093) | 2461 (14.4) | 1946 (11.4) | 9069 (54.6) | 3617 (21.2) |
| ≥25kg/m^2^  (n=4079) | 268 (6.5) | 325 (8.0) | 2099 (51.5) | 1387 (34.0) |
| Total  (n=21172) | 2729 (12.9) | 2271 (10.7) | 11168 (52.8) | 5004 (23.6) |

Values are expressed as number and percentage (%).

There were differences in the proportion of blood pressure levels by BMI in all final stages (p-value<0.01).

BP = blood pressure, HTN = hypertension, BMI = body mass index
